# What’s in a (Sub)strain?

**DOI:** 10.1016/j.stemcr.2018.07.011

**Published:** 2018-08-14

**Authors:** Jill M. Goldstein, Amy J. Wagers

**Affiliations:** 1 Department of Stem Cell and Regenerative Biology, Harvard University, Cambridge, MA 02138, USA; 2 Harvard Stem Cell Institute, Harvard University, Cambridge, MA 02138, USA; 3 Joslin Diabetes Center, Boston, MA 02215, USA

## Abstract

C57BL/6J and C57BL/6N inbred mice are widely, and often interchangeably, used for stem cell research; yet, these substrains harbor discrete genetic differences that can cause phenotypic disparities. In this issue of *Stem Cell Reports*, Morales-Hernández et al. identify one particular difference—disruption of *Nicotinamide Nucleotide Transhydrogenase* (*Nnt*)—that increases reactive oxygen exposure and impairs hematopoietic progenitor cell function in C57BL/6J, as compared to C57BL/6N, mice.

## Main Text

The laboratory mouse has been a highly valuable tool for biological and biomedical research, and the inbred C57BL/6 strain is one of the most widely used across the globe, thanks in part to its fecundity, lifespan, and relatively low predisposition to spontaneous tumors. The origins of the C57BL/6 strain date back to the early 20^th^ century, when Miss Abbie Lathrop began breeding mice on her farm in Granby, Massachusetts. It was there that Lathrop established and maintained several colonies of mice, some of which she later sold to the Harvard geneticist William Castle. In 1921, Castle’s student, C.C. Little (who later went on to found the Jackson Laboratory), crossed female mouse 57 to male mouse 52 from Lathrop’s stocks to generate progeny termed C57 Black (C57BL). Little continued propagating these mice until he established the C57BL/6J substrain at the Jackson Laboratory in 1948. In 1951, a colony of C57BL/6J mice was transferred to the National Institutes of Health (NIH), establishing the C57BL/6N substrain, which was later distributed to several companies including Charles River Laboratories (C57BL/6NCrl), Harlan Sprague Dawley (C57BL/6NHsd), and Taconic Farms (C57BL/6NTac).

**Figure 1:**
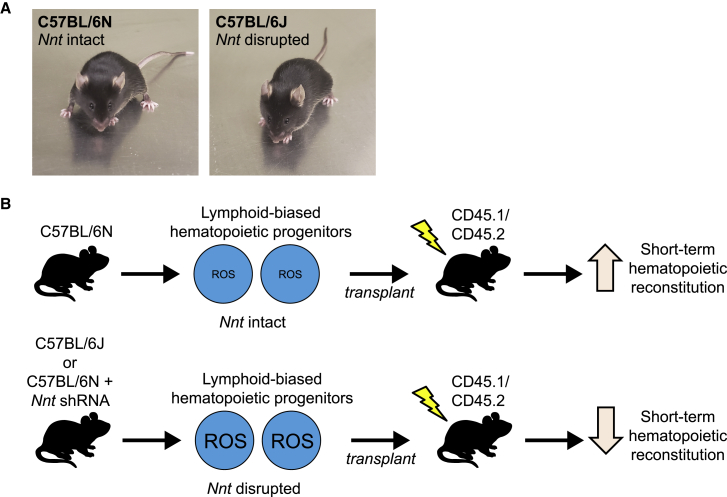
C57BL/6 Substrain-Specific Genetic Differences in *Nnt* Expression Influence Short-Term Blood Repopulation after Hematopoietic Stem and Progenitor Cell Transplantation (A) 2-month-old C57BL/6N (left) and C57BL/6J (right) mice. C57BL/6N mice possess an intact *Nnt* gene, whereas *Nnt* is disrupted in C57BL/6J. (B) Hematopoietic progenitors from C57BL/6N mice (top) exhibit lower ROS levels and increased short-term blood cell reconstitution after transplantation relative to C57BL/6J mice (bottom). shRNA-mediated knockdown of *Nnt* in C57BL/6N hematopoietic progenitors (bottom) recapitulates the increased ROS levels and impaired hematopoietic cell repopulation after transplantation observed in the C57BL/6J substrain.

Now, nearly a century after that fateful 57 × 52 cross, both the C57BL/6J and C57BL/6N substrains serve frequently as “wild-type” controls for scientific inquiry and discovery in individual laboratories and global consortia. The Mouse Genome Sequencing Consortium and the Allen Brain Atlas, for example, chose C57BL/6J as their reference substrain, while the International Knockout Mouse Consortium (now known as the International Mouse Phenotyping Consortium) has disrupted tens of thousands of genes in the C57BL/6N background. In the minds of many investigators, these physically indistinguishable substrains (Figure 1A) are considered essentially interchangeable; however, after generations of breeding in isolation, spontaneous mutations and genetic drift have introduced distinct genetic and phenotypic characteristics within each substrain. For example, C57BL/6N mice harbor mutations in *Crb1* and *Cyfip2*, resulting in photoreceptor degeneration and a lowered cocaine response, respectively (Kumar et al., 2013, Mattapallil et al., 2012). In this issue of *Stem Cell Reports*, Morales-Hernández and colleagues demonstrate that such phenotypic differences among C57BL/6 substrains extend also to critical aspects of stem and progenitor cell biology (Morales-Hernández et al., 2018), revealing how one particular genetic difference (deficiency in the J substrain of the *Nicotinamide Nucleotide Transhydrogenase* [*Nnt*] gene) significantly alters hematopoietic stem and progenitor cell (HSPC) frequency and function in C57BL/6N versus C57BL6/J animals.

Nnt is an enzyme that localizes to the inner mitochondrial membrane and functions to eliminate reactive oxygen species (ROS). In the C57BL/6J substrain, a deletion involving exons 7–11 of *Nnt* results in complete absence of Nnt protein (Huang et al., 2006) and reduced ROS scavenging activity (Ronchi et al., 2013). As prior studies have implicated elevated ROS levels in disrupting HSPC function (Kohli and Passegué, 2014), Morales-Hernández et al. hypothesized that the *Nnt* mutation in C57BL/6J could have functional consequences for hematopoiesis in the two C57BL/6 substrains. To test this hypothesis, the authors first performed *in vivo* competitive transplantation assays using donor bone marrow from the C57BL/6J versus C57BL/6N substrain. Strikingly, recipients reconstituted with HSPCs from C57BL/6N donors exhibited superior donor chimerism for 12 weeks after transplant. The authors traced this enhanced hematopoietic reconstitution capacity to increased repopulation of common lymphoid progenitor (CLP) cells, which boosted production of mature T and B lymphocytes in recipients of C57BL/6N HSPCs. Interestingly, these substrain-specific differences in hematopoietic reconstitution were no longer statistically significant at 16 weeks after transplantation, indicating that the C57BL/6J repopulation defect affects primarily short-term reconstituting, rather than long-term reconstituting, HSPCs.

To clarify the cellular mechanism underlying impaired short-term reconstitution by C57BL/6J HSPCs, Morales-Hernández and colleagues next isolated and transplanted into sub-lethally irradiated recipients 5 different subsets of HSPCs from C57BL/6N or C57BL/6J mice: Long-Term (LT)-HSCs, Short-Term (ST)-HSCs, and three different lineage-biased populations of multipotent progenitor cells (MPP2, MPP3, and MPP4). Analysis of hematopoietic reconstitution via peripheral blood cell chimerism revealed that C57BL/6N-derived MPP3 and MPP4 cells consistently showed greater frequencies of donor cell reconstitution relative to their C57BL/6J counterparts, suggesting that increased functional activity of the lymphoid-biased MPP4s, which are also relatively more abundant than other MPP populations (Pietras et al., 2015), likely explains the superior lymphoid and overall reconstitution by C57BL/6N marrow.

Given the known deletion in *Nnt* in the C57BL/6J substrain and prior work demonstrating functional deficits in HSPCs from mice with overabundant ROS, the authors next tested whether the defects in short-term hematopoietic reconstitution they observed in the C57BL/6J substrain might be due to impaired ROS clearance in *Nnt* mutant HSPCs. Using a fluorescent probe to monitor ROS levels, the authors found that C57BL/6J-derived CLPs indeed exhibited higher ROS levels than C57BL/6N-derived CLPs following transplantation. Furthermore, experiments using polyinosinic:polycytidylic acid (pI:pC) to increase oxidative stress revealed that C57BL/6J HSPCs exhibited higher ROS levels in *in vivo* transplantation assays and *ex vivo* colony formation assays. Finally, the authors linked their findings of increased ROS back to the *Nnt* deficiency within the C57BL/6J substrain. Using shRNAs to suppress *Nnt* in C57BL/6N HSPCs (thereby mimicking the *Nnt* deficiency of the C57BL/6J substrain), the authors showed that *Nnt* knockdown in C57BL/6N HSPCs recapitulated the impaired short-term hematopoietic reconstitution activity and increased ROS levels observed with C57BL/6J HSPCs. Taken together, these findings support the conclusion that *Nnt* disruption in C57BL/6J mice leads to impaired ROS clearance and compromised short-term reconstituting HPSC function, particularly within the lymphoid compartment, after transplantation (Figure 1B).

The novel findings reported by Morales-Hernández et al. raise several issues of significance for the stem cell community. For one, they strengthen accumulating evidence supporting the critical need for stem and progenitor cells to manage cellular oxidative stress for optimal tissue regenerative function. They further indicate a potentially distinct reliance of specific stem/progenitor cell subsets on particular mechanisms of ROS scavenging. Such considerations should be taken into account when choosing substrain background in future studies of oxidative signaling and metabolism.

Finally, and arguably most importantly, the results of Morales-Hernández et al. emphasize the critical importance of careful and transparent accounting of mouse substrains used in studies employing inbred models, an issue that has not previously been addressed specifically for stem and progenitor cell biology. Appropriate control of substrain-specific, as well as strain-specific, genotypic and phenotypic differences is clearly essential to avoid misinterpreting differences in genetic background as consequences of the particular conditions being interrogated. Of note, a recent survey suggested that more than half (!) of published scientific reports using genetically modified mice fail to specify which C57BL/6 substrain was used (Fontaine and Davis, 2016). Issues of substrain variance and incomplete reporting clearly can confound comparisons of results obtained using the “same” genetic model in different labs or over time, and may be particularly acute for long-established and broadly utilized mouse lines, which have been bred and traded among different laboratories, each having potentially distinct substrain preferences for breeding. While Morales-Hernández et al. focused in their current study on HSPCs, given the well-documented effects of oxidative stress on tissue stem cells across organ systems (Chaudhari et al., 2014), it is highly likely that altered ROS scavenging due to *Nnt* deficiency in C57BL/6J mice impacts observations of stem cell functionality in many other cell lineages and regenerative systems. It is also quite likely that additional sequence differences of the >200 identified variations that distinguish C57BL/6J from C57BL/6N mice (Simon et al., 2013) could alter stem cell biology and regenerative capacity in animals of these two substrains. These possibilities should be considered and investigated in future studies.

In conclusion, as unknown or uncontrolled substrain differences have the potential to impact any experiment where mixed genetic compositions of “control” and “experimental” genotypes could unintentionally confound research results, the results of Morales-Hernández et al. mark an important “call to action” for both researchers and scientific journals to increase their efforts to ensure proper selection and reporting of mouse strains and substrains in scientific publications and to review the detailed genetic compositions of their own “C57BL/6” mice.
